# Natural dielectrics for organic field effect transistors: a study on resins derived from larch, spruce and Atlas cedar Pinaceae trees

**DOI:** 10.1039/d5ma00401b

**Published:** 2025-06-18

**Authors:** Corina Schimanofsky, Andreas Petritz, Boyuan Ban, Cristian Vlad Irimia, Rosarita D’Orsi, Cigdem Yumusak, Felix Mayr, Yasin Kanbur, Sunwoo Kim, Alessandra Operamolla, Klara Saller, Manuela Schiek, Yolanda Salinas, Oliver Brüggemann, Christian Teichert, Chunlin Xu, Bong Sup Shim, Clemens Schwarzinger, Barbara Stadlober, Niyazi Serdar Sariciftci, Mihai Irimia-Vladu

**Affiliations:** a Johannes Kepler University Linz, Institute of Physical Chemistry, Linz Institute for Organic Solar Cells (LIOS) Altenberger Str. 69 Linz 4040 Austria mihai.irimia-vladu@jku.at; b Joanneum Research Materials, Institute for Surface Technologies and Photonics Franz-Pichler Str. 30 Weiz 8169 Austria; c Chair of Physics, Department of Physics, Mechanics, and Electrical Engineering, Montanuniversität Leoben Franz Josef Str. 18 8700 Leoben Austria; d Institute of Solid-State Physics, Hefei Institute of Physical Science, Key Lab of Photovoltaic and Energy Conservation Materials Hefei China; e Department of Chemistry and Industrial Chemistry, University of Pisa via Moruzzi 13 56124 Pisa Italy; f Department of Chemistry, Karabük University Baliklarkayasi Mevkii Karabük 78050 Turkey; g Department of Chemical Engineering, Inha University 100 Inha-ro, Michuhol-gu Incheon 22212 Republic of Korea; h Institute for Chemical Technology of Organic Materials, Johannes Kepler University Linz Altenberger Str. 69 Linz 4040 Austria; i Johannes Kepler University Linz, Center for Surface and Nanoanalytics (ZONA) Altenberger 69 4040 Linz Austria; j Institute of Polymer Chemistry, Johannes Kepler University Linz Altenberger 69 4040 Linz Austria; k IMC Krems University of Applied Sciences, Institute of Applied Chemistry Piaristengasse 1 3500 Krems Austria; l Laboratory of Natural Materials Technology/Wood and Paper Chemistry, Åbo Akademi University Porthansgatan 3-5 20500 Åbo Turku Finland

## Abstract

Three Pinaceae resins originating from trees of high industrial significance—European larch, European spruce, and Atlas cedar—were examined in this work. These resins exhibited ease of processing using ethyl alcohol solutions, exceptional film formation, and great dielectric qualities with measured breakdown fields in the range of 5–7.3 MV cm^−1^. Because their film surface was essentially trap-free, it was possible to fabricate organic field effect transistors that are hysteresis-free and have outstanding stability under 12-hour bias stress at working voltages below 10 V, with current retention approaching 90% of the original value and transfer curve recovery occurring within 90 minutes. These environmentally friendly materials, which are freely available, are a great option for applications aiming to produce sustainable electronics.

## Abstract 2

Three naturally occurring resins originating from Pinaceae trees, *i.e.*, spruce, Atlas cedar and larch, are demonstrated to be excellent dielectric materials for organic field effect transistors operating at low voltage.

## Graphical abstract

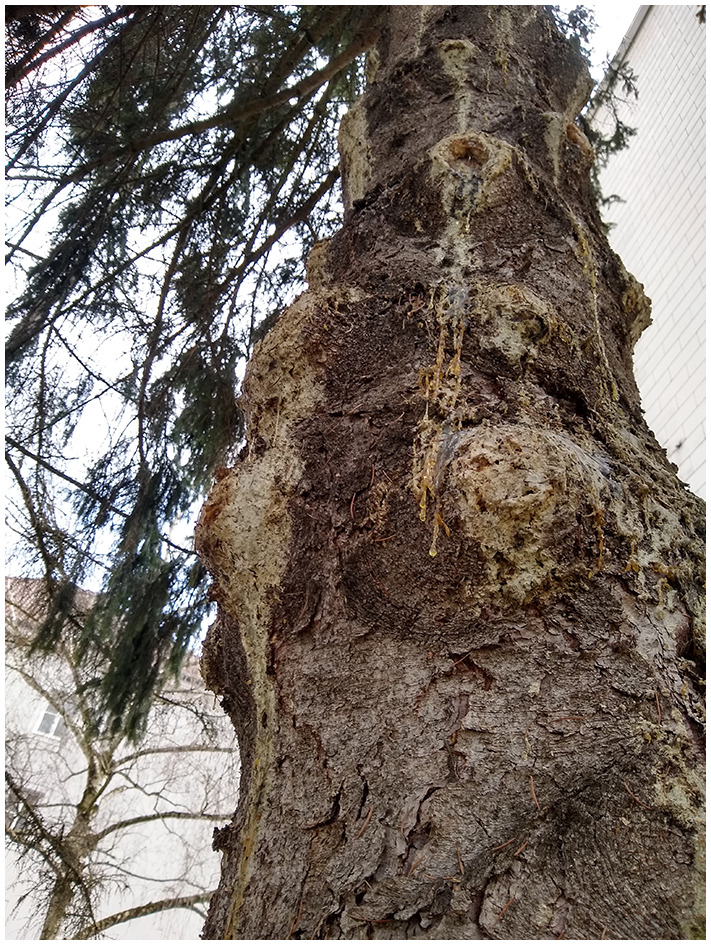

## 1. Introduction

Despite the industrial and commercial potential of organic materials in applications such as organic and perovskite photovoltaics, with reports of efficiencies exceeding 20%, or even light emitting diodes, the development of organic field effect transistors lags behind that of the two counterparts mentioned above. This is mainly due to the low field effect mobility of the employed organic semiconductors and their limited stability and performance when operated in the radio frequency (RF) regime.^1–11^ Although the field of synthetic chemistry has contributed to the tremendous advancement in the performance of organic semiconductors in the recent past,^3,12–14^ many other challenges in terms of materials and their performance remain to be addressed. In this respect, the development of novel substrate and packaging materials, dielectrics and processing conditions are topics of utmost interest for materials scientists.^15–20^ Because of their inherent softness and flexibility, which match the elastic modulus of living cells, organic electronic component materials seem to be perfectly suited for applications interfacing electronics and sensors with living systems and developing disposable diagnostic and drug-delivery technologies.^21–40^ In these applications, fast recording and data transmission speeds are not essential requirements. In order to lessen the environmental impact of e-waste, there is a significant need for sustainable waste management solutions as well as green and biodegradable materials owing to the rapidly expanding application of electronic and sensor devices.^41–45^ In the recent studies on flexible, conformable, and even imperceptible substrates, organic field-effect transistors (OFETs) and organic electrochemical transistors (OECTs) have been explored as viable electrical devices for a variety of applications.^46–50^ In general, OFETs consist of an organic semiconducting and organic dielectric layer sandwiched between three electrodes (gate, source and drain).^51^ Traditional FETs usually employ inorganic materials such as silicon dioxide (SiO_2_),^52^ aluminum oxide (Al_2_O_3_)^53,54^ and other metal oxides (such as titanium, hafnium, tungsten, and tantalum oxides) as the insulating layer.^55^ Despite the inherently higher performance of inorganic dielectric layers, mostly due to the higher purity and order provided by the strong covalent bonds between their component atoms,^56^ the scientific community is becoming increasingly interested in creating OFETs using organic materials that are environmentally benign (even biodegradable), non-toxic, renewable and inexpensive and enable easy processing at low temperatures.^57–61^ Thus, in this work, we complement the efforts by the scientific community in the direction of sustainable electronics production by demonstrating that three natural resins stemming from the widely exploited, industrially relevant Pinaceae trees, *i.e.*, larch, Atlas cedar and spruce, are excellent dielectrics to consider for the fabrication of environmentally friendly electronics.

**Fig. 1:**
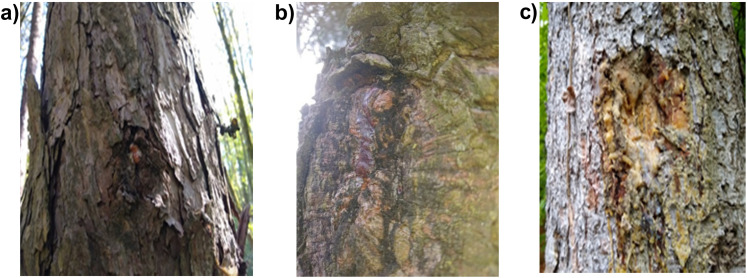
Photograph of the original deposit of the resins analyzed in this study: (a) larch; (b) Atlas cedar; and (c) spruce.

The Atlas cedar (*Cedrus atlantica*) resin reported herein was collected from a living tree growing in the city of Graz, Austria at an altitude of *circa* 350 m. The original resin deposit was captured in the photograph in Fig. 1. The tree from which the resin was collected was an ornamental cultivar. *Cedrus atlantica* is considered to have its origins from the Atlas Mountains in Northern Africa (Morocco) and is widespread around the Mediterranean basin, being closely related to the Lebanese cedar (*Cedrus libani*).^62^ However, Quai *et al.*^63^ argued based on the Phylogenies dataset analysis of *Cedrus* constructed from cpDNA, mtDNA and the combined cp- and mt-DNA that *Cedrus atlantica* could have originated in the high altitudes of Eurasia, and that *Cedrus* migrated into North Africa in the very late Tertiary Period. Many plantations of Atlas cedar are intentionally developed in Europe for timber production.^62,64^ Due to its majestic-round crown, the Atlas cedar is also intentionally planted in many city parks and residential areas for esthetic reasons.

The European larch (*Larix decidua*) resin reported herein was collected from a cultivar tree growing in the forest around Joanneum Research Materials, Weiz, Austria, at an altitude of *circa* 570 m, with the original resin deposit being captured in the photograph in Fig. 1. European larch is a fast-growing deciduous coniferous tree, which is mainly found in the southern, eastern and central mountains of Europe.^65^ It can be divided into three groups according to its geographic distribution including Alpine larch (*Larix decidua* var. *decidua*), Carpathian larch (*Larix decidua* var. *carpatica*), and Poland larch (*Larix decidua* var. *polonica*). Larch trees can grow up to approximately 50 meters with a lifespan of up to a couple of thousand years, being in fact the tallest of all Pinaceae trees.^66,67^ Owing to their strong adaptability, avoiding foliage desiccation by losing their needles in the winter in the harsh cold environment, larch trees have also been planted in other areas, such as north-western Europe, Siberia, Japan, North America, and New Zealand. Considering that larch wood has high durability, it is often used as timber and pulp fibers for manufacturing furniture and paper, respectively. Moreover, attempts have been made to valorize other components in addition to fibers. For example, as side products to pulp and paper, tannins can be extracted from bark and resins from wood. Distillation of larch resin produces turpentine, which is used as traditional medicine for treating colds.^68^

The European spruce (*Picea abies*) resin reported herein was collected from a cultivar tree growing in the forest around the Mariatrost neighborhood in the city of Graz, Austria, at an altitude of *circa* 405 m. The original resin deposit was captured in the photograph in Fig. 1. The Latin name *abies* means “fir-like” in the sense that it refers to it being tall., *i.e.*, fir-like tall. The European spruce is widely planted for wood and represents the most prevalent tree in Eurasia, filling more than 30 million hectares of forest land, or more than 55% of the entire forest area of Eurasia.^69^ The reason for this widespread distribution is the re-plantation of the European forests over the past centuries. Because of this re-plantation, nowadays spruce occupies more than 20% of the land outside its native range, primarily on the land of broadleaved forest sites at low altitudes.^70^*Picea abies* is also used as the main Christmas tree in several countries around the world. Importantly, spruce also represents the first gymnosperm to have its genome sequenced.^71^

## 2. Experimental

The resins employed in this work were collected from living trees in Austria, Europe. The resins were solubilized in pure ethanol (99.9%) in 0.1 g mL^−1^ concentration by heating the solution while stirring for 30 min at 50 °C. The solution was filtered through a Chromafil, 0.2 μm pore size hydrophilic filter paper, and then diluted to the concentration employed for depositing thin films in this work, *i.e.*, 25 mg mL^−1^ or 20 mg mL^−1^, respectively. All the resin thin films investigated in this work were spin coated at a rotation speed of 2500 rpm, and then dried on the top of a hot plate in air for 1 h at a temperature not exceeding 80 °C.

A TGA/PerkinElmer Q5000 instrument at the Institute of Polymer Chemistry, Johannes Kepler University Linz, Austria was used to perform thermogravimetric tests utilizing platinum pans and scanning from 70 °C to 900 °C at a heating rate of 10 °C min^−1^ in a nitrogen environment (25 mL min^−1^). Using the same experimental heating setup and protocol, all four pine resin samples (in amounts ranging from 5 to 15 mg) were examined. The content of each resin material was taken from its corresponding pellet (nugget).

We performed gas chromatography measurements for the three resins in this study by dissolving each resin in 2 mL ethanol and used the solutions for the respective study (usually containing around 200 mg dry resin per sample; however, some solutions contained around 40–60 mg of dry resin). From each solution, 100 μL was withdrawn and transferred into a tared 1.5-mL glass vial with the aid of a microbalance. The solvent was evaporated to dryness using a stream of nitrogen, and then the vials were kept in a vacuum oven (at 40 °C) for *ca.* 30 min. After cooling to room temperature, the vials were weighed for the determination of the dry extract weight. A volume of 1.0 or 1.5 mL of acetone was added, and the vials were kept for *circa* 1 min in an ultrasonic bath. A volume corresponding to around 0.6 mg of each dry resin was withdrawn and transferred to a 6 mL test tube. Then, 2 mL of a solution containing 40 μg each of four internal standards (ISs) was added, and the solvent was evaporated to dryness using a stream of nitrogen in a 40 °C water bath. Subsequently, silylation reagents were added including pyridine-BSTFA-TMCS 20 : 80 : 20 μL, and the tubes were kept in an oven at 70 °C for 30 min, where BSTFA-TMSC stands for *N*,*O*-bis-(trimethylsilyl)-trifluoroacetamide with trimethylchlorosilane. After this, the solutions were transferred to 1.5-mL GC vials using Pasteur pipettes with a glass insert. Then approximately 1 μL of each sample was injected to the GC-MS. Identification was performed by comparing the mass spectra with that in spectra databases including NIST12/Wiley 11th and our own database (*i.e.*, ABÖ Akademi, Finland). The peak areas were integrated and the concentration of each compound was calculated by dividing the peak area with that of the IS (heneicosanoic acid for all compounds was eluted before cholestadiene, and cholesterol or betulinol for cholestadiene and lignans). Then, the result was multiplied by the added amount of IS, and the result was divided by the amount of dry resin taken for the analysis.

To gain a better understanding of the three resin compositions, in addition to gas chromatography, we also performed HPLC high-resolution MS analyses (HPLC-MS) of all the samples and pyrolysis-gas chromatography-mass spectrometry (pyrolysis-GC-MS) analysis, given that this technique does not discriminate the insoluble fractions of the resins. The molar mass distribution of the resins was obtained by size exclusion chromatography. In the case of HPLC-MS, roughly 10 mg of the sample was mixed with 1 mL of acetonitrile and sonicated for 5 min, centrifuged and the soluble fraction analyzed. The characterization of resin compounds was performed using a Thermo Scientific Surveyor HPLC system coupled to a LTQ Orbitrap Velos mass spectrometer, available at the Institute for Chemical Technology of Organic Materials, Johannes Kepler University Linz, Austria. The compounds were separated on a Thermo Scientific Accucore C18 column (150 mm × 3.0 mm, 2.6 μm particle size) using a gradient with mobile phase A containing 0.1% formic acid in water and mobile phase B containing 0.1% formic acid in acetonitrile, at a flow rate of 0.5 mL min^−1^. The elution gradient starting conditions were 95% A and 5% B. After 2 min of equilibration time, the proportion of B was increased to 20% at 8 min, to 40% at 12 min, to 60% at 15 min and to 95% at 19 min, and held constant for another 4 min. UV detection was done by a photodiode array detector and mass spectra were recorded with an atmospheric pressure chemical ionization interface in FT mode with a resolution of 30 000. In pyrolysis-GC-MS, to achieve a better performance in the analysis of natural products, pyrolysis in the presence of tetramethylammonium hydroxide (also known as thermally assisted hydrolysis and methylation) was performed. Experiments were carried out with a CDS Pyroprobe 5250 pyrolyzer (CDS Analytical Inc.) coupled to a Trace GC Ultra (Thermo Electron Corp.) equipped with a Restek RTX35 (30 m × 0.32 mm × 0.25 μm) capillary column, and an MD 800 quadrupole mass spectrometer (Fisons Instruments), available at the Institute for Chemical Technology of Organic Materials, Johannes Kepler University Linz, Austria. Briefly, 5 μL of a saturated aqueous tetramethylammonium hydroxide (TMAH, Fluka) solution was added to about 100 μg of sample and pyrolysis was performed at 550 °C for 10 s. The pyrolizer interface was set at 300 °C and the injector at 280 °C. The GC column temperature conditions were as follows: initial temperature 50 °C, hold for 2 min, increase at 20 °C min^−1^ to 300 °C, and hold this temperature for 10 min. The helium gas flow was set to 0.8 mL min^−1^ and the split flow was 14 mL min^−1^. Mass spectra were recorded under electron impact ionization at 70 eV electron energy in the *m*/*z* range of 15–400. Identification of the compounds was done by comparison of their mass spectra with the NIST 2011 electronic library and literature. In size exclusion chromatography, the samples were mixed with tetrahydrofuran (THF) and the insoluble part filtered off. Separation was carried out using three Phenomenex Phenogel columns (300 × 4.6 mm) with pore sizes of 50, 500 and 1000 Å at a temperature of 40 °C under a THF flow of 0.35 mL min^−1^. Detection was done using a refractive index and a UV detector, and for calibration, a set of polystyrene standards was used.

Phosphitylation of samples (*i.e.*, ^31^P-NMR spectra) was performed adapting the method described elsewhere.^72^ The samples were dried overnight in an oven set at 40 °C, and then transferred to a desiccator until they reached room temperature. A mixture of pyridine and deuterated chloroform (CDCl_3_) with the ratio 1.6 : 1 v : v was prepared and dried over molecular sieves. Using this mixture, a 0.1 M solution of the relaxation reagent, chromium(iii) acetylacetonate (5 mg mL^−1^), and the internal standard, cholesterol (40 mg mL^−1^), was prepared. All the solutions were stored in the dark. About 40 mg of sample was dissolved in 0.5 mL of solvent solution in a vial equipped with a stirring bar. Then, 0.1 mL of the internal standard and relaxation solution was added, and the solution was stirred for ∼4 h to achieve solubilization of the sample. 0.1 mL of 2-chloro-4,4,5,5-tetramethyl-1,3,2-dioxaphospholane (TMDP) was added to the clear solution and it was kept under vigorous magnetic stirring for 30 min. The resulting solution was transferred into a NMR tube. ^31^P-NMR spectra were recorded on a JEOL YH spectrometer with a probe operating at 202.468 MHz at 25 °C, available at the Department of Chemistry and Industrial Chemistry of University of Pisa, Italy. Chemical shifts were calibrated using the ^31^P-NMR signal at 132.2 ppm arising from the reaction product between residual water and TMDP. The spectra were quantitative and proton broadband decoupling was applied during the acquisition time. Cholesterol was used as an internal standard. Spectra were acquired with a 100 ppm spectral width, 32 000 data points, 11 s relaxation delay, and 256 scans. The spectra were analysed using the JEOL Delta software.

Attenuated total reflection Fourier-transform infrared (ATR-FTIR) spectra were measured on a Bruker Vertex 80 FTIR spectrometer equipped with a Bruker Platinum ATR unit and a liquid N_2_-cooled mercury cadmium telluride (MCT) detector (at the Institute of Physical Chemistry, Linz Institute for Organic Solar Cells, of Johannes Kepler University Linz, Austria). Spectra were recorded with a resolution of 1 cm^−1^ and averaging of 200 scans. The solid resin material for ATR-FTIR measurements was obtained from ethanolic solutions of the resins by depositing the resin on glass substrates *via* drop-casting, drying the resulting film at 80 °C and carefully scraping off the resin material from the substrate.

Atomic force microscopy (AFM) and Kelvin probe force microscopy (KPFM) measurements were performed using an Asylum Research MFP-3DAFM system available at the Department of Physics of Montan University Leoben, Austria. ASYELEC-01-R2 probes were used (Ti/Ir coating on both the reflective and tip sides of the cantilever, spring constant of ∼2.8 N m^−1^, resonant frequency of ∼75 kHz, and tip radius of 25 ± 10 nm). For both types of experiments, the resin was deposited by spin coating from an ethanol solution on a gold-coated glass slide, and during the measurement, the gold back electrode of the resin films was grounded. AM-KPFM measurements providing contact potential differences (CPD)^73^ were carried out in a two-pass mode, with the probe lifted by 10 nm in the second pass. The root mean square (RMS) data was proven for both the topography roughness and the CPD fluctuations as the average with the standard deviation considering five arbitrarily chosen 20 × 20 μm^2^ areas of each resin sample. Topography and CPD images were processed in the open-source software Gwyddion v2.62. In the case of the topography images, first-order line filtering was applied and leveling of the base plane. In the CPD images, only zero-order line filtering was applied.

Contact angle measurements conducted at Joanneum Research Materials, on a KRÜSS DSA 100 contact angle measuring system, were performed to determine the surface energy. The liquids used were ultrapure water (a very polar liquid with a surface tension *γ* = 72.8 mN m^−1^ separable in a polar component *γ*^P^ = 51 mN m^−1^ and a dispersive component *γ*^D^ = 21.8 mN m^−1^), and diiodomethane (a very nonpolar liquid with *γ* = 50.8 mN m^−1^, *γ*^P^ = 0 mN m^−1^ and *γ*^D^ = 50.8 mN m^−1^). The surface energy was calculated from five droplets of water and diiodomethane, respectively, for each of the resins, and the mean values from three droplets with their standard derivations are reported herein.

In the dielectric strength measurement (breakdown field), the resin was measured in the metal–insulator–metal (MIM) configuration. Therefore, the resin was spin-coated on top of a 1 mm wide and 80 nm thick aluminum electrode. The resulting film was dried in ambient air at 80 °C before another 1 mm wide and 80 nm thick aluminum electrode was deposited on top in a cross configuration. The processing of all the resin thin films deposited in this study (*i.e.* deposition and drying) was done in ambient air. The MIM device was connected *via* alligator clips to a Novocontrol impedance analyzer with a DC booster instrument working up to 500 V DC voltage, available at the Institute of Physical Chemistry of Johannes Kepler University Linz, Austria. To establish the breakdown field, the DC voltage scan was performed at an increment of 2 V and 2.5-s time at each applied voltage starting from 0 V. As the breakdown voltage, the voltage considered was the one where a sudden loss of several orders of magnitude in capacitance occurred. The break down field was calculated by dividing the breakdown voltage to the measured thickness of the film *via* profilometry in the immediate vicinity of the measured MIM structure. For the determination of the dielectric constant, we performed impedance spectroscopy between 10 kHz and 1 mHz and used it to calculate the capacitance value at 1 kHz. For that, we employed the spin-coated resins in the MIM structure described above.

In the ellipsometry measurement, resin layers were prepared on fused silica substrates. Therefore, the resin was spin-coated, and subsequently dried at 80 °C. Standard ellipsometry scans to obtain reflection and transmission intensity data were recorded using a J. A. Woollam M 2000 DI ellipsometer in the spectral range of 195 nm to 1685 nm, available at the Center for Surface and Nanoanalytics (ZONA), Johannes Kepler University Linz, Austria. However, the transmission measurements were limited by the fused silica substrate given that it is only sufficiently transparent down to 230 nm. Data were analyzed with Complete EASE starting with a Cauchy layer in the transparent range to fit the layer thickness. Conversion to a transparent B-spline (Kramers-Kronig consistent mode on, 0.2 eV spectral resolution) with automated wavelength expansion fit gave the complex refractive index of the resin layers. Ellipsometric and transmission data were analyzed jointly, and backside reflection was included in the fitting.^74^

The aluminum gate electrode itself has a thickness of 80 nm and was obtained by fast evaporation (rate of 4–5 nm s^−1^) of high purity aluminum wires (99.999%, ChemPUR GmbH). The resulting film was anodized at a voltage of 10 V, while maintaining a steady current of 15 mA. After the 10 V compliance was reached, the sample was allowed to continue anodization until a final current of ∼4.5 μA. The typical thickness of the anodized layer is given by the following equation:*d* = *α*(*V* − *V*_ox_),where *α* is the oxide forming factor (1.6 for aluminum), *V* is the maximum voltage applied, and *V*_ox_ is the voltage necessary to generate the native oxide (∼1.35 V for aluminum). As the organic semiconductor, either pentacene or C_60_ (from Aldrich) was used. The used material was purified twice through sublimation before it was deposited on the device through physical vapor deposition (PVD). The parameters (vacuum level, temperature ramp, deposition rate of 0.2–0.3 Å s^−1^, *etc.*) were adjusted to finally obtain a 60 nm-thick film of the semiconductor. To complete the device, the source and drain electrodes (aluminum for C_60_ and gold for pentacene) were deposited using a physical vapor deposition system in a glove box under nitrogen. Both aluminum and gold were procured from ÖGUSSA, with a purity of 99.999% for aluminum and 99.99% for gold. The OFET samples were measured with a probe station located in a glove box under nitrogen. We fabricated and measured the OFET devices following identical methods and procedures both at the Institute of Physical Chemistry, Johannes Kepler University Linz, Austria and the Joanneum Research Materials, Weiz, Austria.

## 3. Results and discussion

### 3.1 Materials analysis

The photographs of the original resin materials are presented in Fig. 1. A particular case was represented by spruce resin, for which we complemented the OFET study with one more resin sample collected from a tree in the vicinity of the city of Linz (altitude of *ca.* 570 m), Austria. For this particular spruce resin, only a size exclusion chromatography experiment was run for comparison with the original resin collected from the forest surrounding the city of Graz, Austria. The rationale for this decision will be discussed in the OFET section herein.

**Fig. 2:**
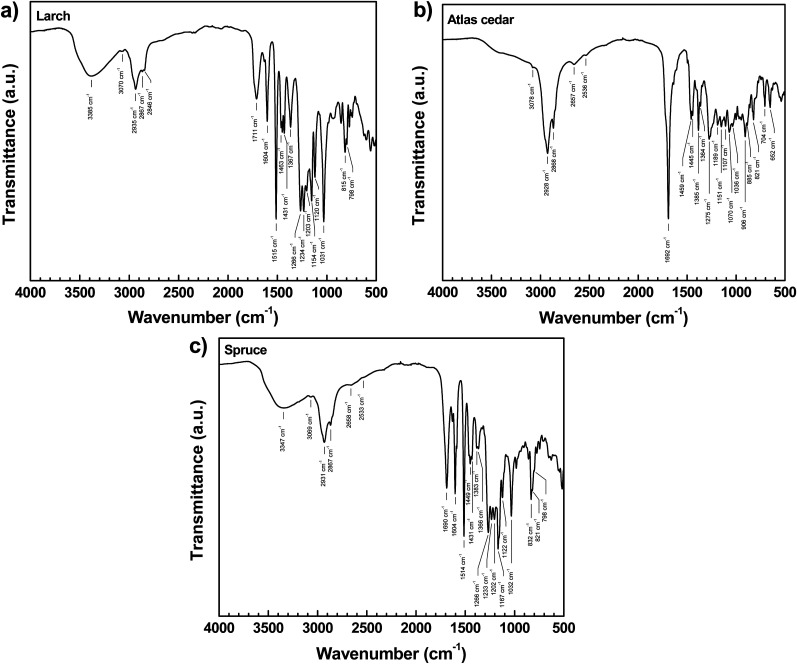
ATR-FTIR spectra of (a) larch, (b) Atlas cedar, and (c) spruce resins. The absorption bands are marked on each graph.

The ATR-FTIR spectra of the investigated resins are shown in Fig. 2a–c. In case of the larch resin (Fig. 2a), the broad absorption band in the ATR-FTIR spectrum, which can be seen at 3385 cm^−1^, is characteristic of the O–H vibration of intermolecularly hydrogen-bonded hydroxyl groups in alcohol or phenol groups. The higher alcoholic group content in larch resin was indeed confirmed by ^31^P NMR spectroscopy, and this aspect will also be discussed in the following section. At lower wavenumbers, other absorption bands are visible, including the C–H stretching vibration modes of aromatic or alkene C–H groups (3070 cm^−1^), as well as the C–H in methylene and methyl groups (2935–2846 cm^−1^).^75^ Additionally, the spectrum exhibits a very broad absorption band in the region of approximately 3600–2400 cm^−1^, which can be assigned to the O–H stretching vibration of hydrogen-bonded carboxylic acid groups. This band appears with a very low relative intensity, and a more accurate evaluation of its intensity is difficult due to its partial overlap with the alcohol or phenol O–H vibration bands. However, considering the low content of free carboxyl groups according to NMR (will be discussed in the following section), the shape of the spectrum looks consistent with the data. The comparatively broad band of medium intensity at 1711 cm^−1^ can be attributed to the carbonyl group (C=O) stretching vibration and indicates the presence of ester and/or carboxylic acid groups.^75^ The sharp and intense bands at 1604 cm^−1^ and 1515 cm^−1^ arise from the aromatic ring C=C stretching vibrations and are typical for substituted phenolic groups such as guaiacyl units (4-hydroxy-3-methoxyphenyl group)^76^ or 4-hydroxyphenyl substituent groups.^77,78^ The spectrum of Atlas cedar (Fig. 2b) shows a broad O–H stretching vibration band (3600–2500 cm^−1^), a sharp C=O vibration (1692 cm^−1^), and overtone vibrations (2657 and 2536 cm^−1^), which can be assigned to the carboxylic acid group of terpenoid acids. However, in the spectrum no characteristic aromatic C=C stretching of phenol derivatives can be found (typically near 1604 and 1515 cm^−1^). This is consistent with the low content of phenols or substituted phenol in the composition determined by GC-MS (to be discussed in the following). The FTIR spectrum of spruce resin (Fig. 2c) is similar to that of larch resin. The spectrum shows a broad band centered at 3347 cm^−1^, which arises from the O–H stretching vibration occurring in alcohols and phenols. Additionally, the presence of a significant content of carboxylic acid groups in the resin is shown by the very broad hydrogen-bonded carboxylic acid O–H stretching vibration in the wavenumber range of 3600–2400 cm^−1^, the carbonyl C=O stretching vibration band at 1690 cm^−1^ and the weak overtone vibration bands at 2658 cm^−1^ and 2533 cm^−1^, confirming the higher concentration of free COOH groups found in these two samples by ^31^P NMR spectroscopy. The strong characteristic C=C stretching bands at 1604 cm^−1^ and 1514 cm^−1^ indicate the presence of compounds with phenolic groups in the resin.^76–78^ The coalescing bands at 832, 821 and 798 cm^−1^ can be assigned to the out-of-plane C–H bending of the different aromatic constituents. The observed bands corroborate the results of GC-MS analysis of the investigated spruce resin and can be related to the occurrence of both phenolic group-containing aromatic compounds (*e.g.*, 4-hydroxycinnamic acid and pinoresinol) and terpenoid acids as the major constituents of the resins.

**Fig. 3:**
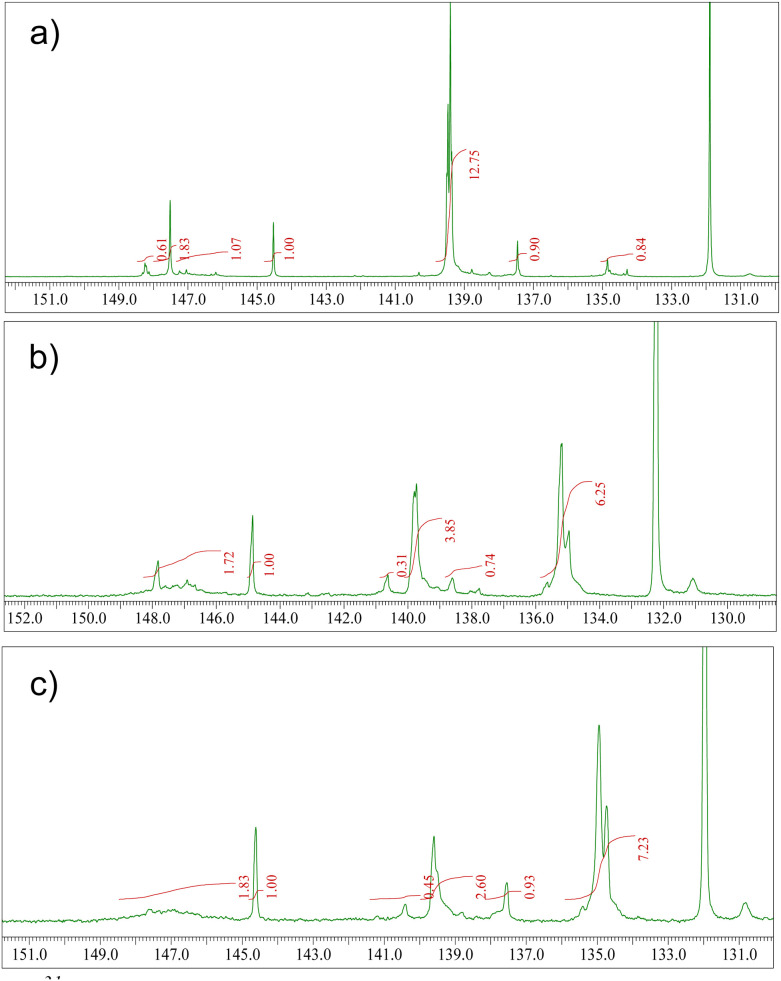
^31^P NMR spectra in the 130–150 ppm region recorded for (a) larch, (b) Atlas cedar, and (c) spruce after derivatization of the resins with TMDP. The 132.2 ppm signal was derived from the reaction of residual TMDP with water and used for the calibration of the chemical shifts.

^31^P-NMR spectra were recorded after derivatization of the resins with 2-chloro-4,4,5,5-tetramethyl-1,3,2-dioxaphospholane (TMDP). The spectra were analyzed in the spectral range of 130 to 150 ppm (Fig. 3). The calculated hydroxyl content, obtained after standardization with a known quantity of cholesterol, is reported as millimole per gram of sample in Table 1. The respective table shows that the spruce and Atlas cedar resins possess a high content of carboxylic groups, in good agreement with the results from mass spectrometry determination of the molecular composition of the three resins (will be presented in the following section). Among the resins, Atlas cedar possessed the greatest content of aromatic compounds, where the major content corresponds to the substituted compound (see Table 1). Larch resin seems to be different from the others because it has more defined peaks in the aliphatic hydroxylic group region, with particular attention to the peak at 147.5 ppm, which can be ascribed to 2-substituted ethanolic groups. Larch has a higher content of aromatic groups, in particular substituted ones, and different from the others it presents a very low content of carboxylic acids. The high content of lignans found in larch resin well explains the high content of substituted aromatic groups revealed by NMR (Fig. 3).

**Table 1:** Calculated hydroxyl content in mmol per gram of the sample for the resins investigated in this study. Integration limits

Resin	Aliphatic OH^a^	Phenolic OH^b^	Substituted phenolic OH^c^	Carboxylic acid^d^
Larch	0.73	0.19	2.66	0.18
Atlas cedar	0.35	0.15	0.86	1.29
Spruce	0.39	0.20	0.65	1.55

a 149.0–145.0 ppm.

b Phenolic hydroxyl content, 138.8–137.4 ppm.

c Content of phenolic hydroxyls with substitution on C5 carbon of the aromatic ring, 144.0–139 ppm.

d 136–133.6 ppm. Integration is referred to as cholesterol used as an internal standard.

The composition of the individual resins was determined through their MS spectra. The species detected in the measurement are listed in Table 2. In our collected Atlas cedar resin, we observed that resin acids were the dominant compound group (24%), with isopimaric acid dominating (8%), followed by dehydroabietic acid (7%) and abietic acid (5%). Overall, diterpenoids accounted for 32% of the resin weight; however, a significant amount (9.0%) of the GC eluted compounds (42% in total) were unidentified. Holmbom *et al.*^79^ characterized callus resin (three approximately one-year-old, separate samples) and found that lariciresinol-9-acetate (up to 19.5%), lariciresinol (up to 12.2%), caffeic acid (up to 7.5%), lariciresinol-coumarate (up to 6.2%), abietol (up to 7.6%), abieta-7,13-diene (up to 4.8%), and lignan esters (up to 3.9%) were the major components among the GC-identified compounds. Unidentified peaks accounted for 16%, while compounds not eluted by GC accounted for 36% of the resin weight. In comparison, the major constituents of larch resin analyzed by us *via* the GC technique are presented as lariciresinol-9-acetate, a derivative of the lignan lariciresinol, which was the dominant component of our larch resin sample, accounting for 18.2% of the resin weight, followed by the underivatised lariciresinol, which accounted for 10.3%. The other lignans present were isolariciresinol (0.26%), which is easily formed from lariciresinol, and pinoresinol (0.77%). This is well in accordance with the result obtained by Holmbom *et al.*^79^ Altogether, the lignans accounted for 29% of the resin weight, and formed the major components of this larch resin sample. In fact, lariciresinol received its name from the *Larix decidua* species. The other major components were caffeic acid (5.8%), also well in accordance with a previous study (Holmbom *et al.*^79^), and the diterpenoids manool and larixol, both accounting for 2.2% of the resin weight. However, the diterpenoid composition was different than that previously reported for larch callus resin (Holmbom *et al.*^79^). The unidentified GC-eluted compounds accounted for only 2.4% of the resin weight, and compounds not eluted on GC for 48%. This means that the identity of almost half of the material is still unknown. Zoubi *et al.*^80^ analyzed the content of Atlas cedar resin and found ∼1.1% content of essential oils. The major components identified in the GC experiment run in this study were α-, β-, and γ-himachalene, which accounted for ∼60% of the resin composition, as well as resin alcohols such as cedrol and isocedranol in the proportion of ∼10% and 5%, respectively. In our collected Atlas cedar resin, we observed that resin acids were the dominant compound group (24%), with isopimaric acid dominating (8%), followed by dehydroabietic acid (7%) and abietic acid (5%). The other detected notable diterpenoids were neoabietic and palustric acid (1.02% and 0.32%, respectively), and isopimarol, manool, isopimaral, and pimarol (2.93%, 1.45%, 0.49%, and 0.12%, respectively). Overall, diterpenoids accounted for 32% of the resin weight; however, a significant amount (9.0%) of the GC-eluted compounds (altogether 42%) were unidentified. The HPLC-HRMS and SEC investigations presented in the following show the full composition of Atlas cedar resin employed in this work, and a list of its constituents is presented in Tables 3 and 4. Similar to the case of Atlas cedar, for the European spruce, also the resin acids were the dominant compound group (15%), with dehydroabietic acid dominating (5.8%), followed by palustric and isopimaric acid (around 2% each). The other noticeable detected diterpenoids were the resin acids abietic, sandaracopimaric, neoabietic, and pimaric acid (1.59%, 1.26%, 1.01%, and 0.49%, respectively) and pimarol (0.14%). However, in European spruce, lignans were also a significant compound group (17%), dominated by pinoresinol (4.4%) and lariciresinols (3.1%). The other detected noticeable lignans were secoisolarici-, matai-, and isolarici-resinol (0.39%, 0.098, and 0.059%, respectively). Among the resins in this study, only larch contained more lignans that spruce. The other important detected aromatic compounds were 4-hydroxycinnamic acid (concentration 8.19%), caffeic acid, ferulic acid, and isoferulic acid (0.26%, 0.18%, and 0.010%, respectively). Cholestadiene accounted for 0.048% of the resin weight. In the case of larch and Atlas cedar, only around 40% of the material was eluted from the GC column in our study. The composition of European spruce resin was analyzed by Holmbom *et al.*,^79^ who obtained very different results for European spruce callus resins (four different resins analyzed in their study), where pinoresinol and *p*-coumaric acid were the dominant compounds with a much higher content (up to 19% and 15%, respectively). Dehydroabietic acid was detected only in minor amounts, and the content of resin acids was overall much lower than in the resin used in the present study, which has more similarities to oleoresin of European spruce, although it contains lignans (Holmbom *et al.*^79^). However, the analysis results are not directly comparable, given that different quantification methods were used in the study presented in ref. 79 compared to the present study.

**Table 2:** Concentration of the species in the resin samples (larch, Atlas cedar, and spruce) detected through MS in mg g^−1^ dry ethanol extract. nd = not detected

Component	Larch (mg g^−1^)	Atlas cedar (mg g^−1^)	Spruce (mg g^−1^)
Mono- and sesquiterpenoids			
β-Phellandrene	nd	nd	nd
Longifolene	nd	nd	nd
Cadinenes (β, δ)	nd	nd	1.18
11-Hydroxy-eremophil-1(10)-ene	nd	nd	nd
α-Terpineol	nd	nd	0.721
α-Terpinolene	nd	nd	0.125
β-Cubebene	nd	nd	0.105
Germacrene D	nd	nd	0.091
**Sum**	**0**	**0**	**2.22**

Diterpenoids			
Secodehydroabietic acids	nd	11.9	nd
Pimaric acid	nd	nd	4.93
Sandaracopimaric acid	0.506	7.63	12.6
Isopimaric acid	nd	81.0	20.4
Abietatetraenoic acid(s)	nd	1.01	0.688
Palustric acid	0.391	3.25	23.9
Dehydroabietic acid (DeAb)	2.71	69.0	57.9
Methyl dehydroabietate	nd	nd	nd
Abietic acid (Ab)	nd	53.4	15.9
Abietapentaenoic acid	nd	nd	nd
Neoabietic acid	nd	10.2	10.1
8,15-Pimaradien-18-oic acid	nd	nd	nd
**Sum**	**3.61**	**237**	**146**

Oxidised compounds			
Hydroxy-DeAbs	nd	5.28	9.26
Hydroxy-Ab(s)	nd	13.0	5.79
Hydroxy-RA	nd	1.92	0.523
Dihydroxy-DeAb(s)	nd	0.385	0.825
Hydroxy-7-oxo-DeAb	nd	nd	0.507
**Sum**	**0**	**20.6**	**16.9**

Other diterpenoids			
Thunbergene(s)	nd	nd	0.683
Thunbergol	nd	nd	nd
*Cis*-abienol	nd	nd	3.61
Isopimaradiene	nd	1.09	nd
19-Norabieta-8,11,13-triene	nd	0.275	nd
Abieta-7,13-diene	0.381	0.678	nd
Manool	21.9	14.5	nd
Manool oxide	nd	nd	0.363
Pimaral	nd	nd	nd
Isopimaral	nd	4.86	nd
Palustral	nd	nd	nd
Dehydroabietal	nd	2.49	nd
Pimarol	nd	1.20	1.37
Isopimarol	10.6	29.3	nd
Palustrol	nd	nd	0.949
Abietal	2.40	3.24	nd
Dehydroabietol	0.795	7.11	0.788
Neoabietol	nd	0.661	0.523
Larixol	21.7	nd	nd
**Sum**	**57.8**	**65.4**	**8.29**

Small-molecular aliphatic acids			
Lactic acid	4.56	3.03	1.72
Glycolic acid	nd	1.02	nd
3-Hydroxypropanoic acid	nd	0.521	nd
Succinic acid	nd	0.422	0.492
Levulinic acid	nd	nd	nd
2-Methyl-4-oxopentanoic acid	nd	nd	nd
Methylsuccinic acid	nd	nd	nd
**Sum**	**4.56**	**4.99**	**2.21**

Fatty acids and alcohols			
*n*-Octanoic acid	nd	nd	nd
*n*-Nonanoic acid	nd	0.207	nd
*n*-Hexadecanoic acid	0.896	1.14	0.589
*n*-Heptadecanoic acid	nd	nd	nd
*n*-Octadecanoic acid	nd	nd	nd
*n*-Hexadecanol	0.258	nd	0.892
*n*-Octadecanol	0.265	nd	0.712
**Sum**	**1.42**	**1.35**	**2.19**

Aromatic compounds			
Resorcinol	nd	nd	0.284
Vanillin	3.50	nd	nd
Cinnamic acids	0.318	nd	nd
*p*-Coumaric acid	nd	nd	nd
4-Hydroxy-benzaldehyde	nd	nd	0.512
3,4-Dihydroxybenz-aldehyde	0.978	nd	nd
3-Hydroxy-4-methoxybenzaldehyde	nd	nd	nd
Vanillic acid	0.323	nd	nd
3-Hydroxybenzoic acid	nd	nd	0.176
3,4-Dihydroxybenzoic acid	0.183	nd	nd
Vanillic acid	nd	nd	0.417
Ferulic acids	2.27	nd	1.77
Isoferulic acid	nd	nd	0.103
4-Hydroxycinnamic acid	3.82	nd	81.9
3,4-Dihydroxycinnamic acid	57.6	nd	2.61
1-Guaiacylglycerols	nd	nd	nd
Monomethyl pinosylvin	nd	nd	nd
Isolariciresinol	2.60	nd	0.593
Secoisolariciresinol	nd	nd	3.93
Lariciresinol	103	nd	13.3
Matairesinol	nd	nd	0.983
Lariciresinol-9-acetate	182	nd	nd
Pinoresinol	7.66	nd	43.8
7′-Oxolariciresinol (?)	nd	nd	1.04
Lariciresinol coumarates	nd	nd	18.4
**Sum**	**364**	**0**	**170**

Miscellaneous			
Carbonic acid	nd	nd	nd
Glycerol	1.47	0.541	0.630
Cholestadiene	0.734	nd	0.476
Diacetone alcohol	6.02	0.303	1.14
*N*,*N*-Diethylcarbamic acid	0.327	0.419	nd
Ethylene glycol	1.50	nd	0.865
Pentitol	nd	nd	0.268
Hexitol	0.407	nd	nd
Monopalmitin	0.402	nd	nd
Decalin derivative	11.1	nd	nd
**Sum**	**21.9**	**1.26**	**3.38**
**Identified, sum**	**453**	**331**	**351**
**Unidentified peaks**	**24**	**90**	**41**
**% GC eluted**	**47.7%**	**42.1%**	**39.2%**

**Table 3:** HPLC-HRMS analysis of the acetonitrile-soluble fraction of the resins

*t*_R_ (UV)/min	MH^+^	Name/sum formula	Atlas cedar	Larch	Spruce
			% area (UV)		
6.15	181.0493	C_9_H_8_O_4_	0.51	1.89	—
8.17	165.0544	Hydroxycinnamic acid	—	1.24	13.37
9.24	195.065	C_10_H_10_O_4_	—	—	0.28
9.93	377.1593	C_20_H_24_O_7_	—	0.5	—
11.47	375.1438	C_20_H_22_O_7_	—	0.51	0.31
11.58	327.1588	C_20_H_22_O_4_	—	0.58	0.24
11.92	219.1014	C_13_H_14_O_3_	2.28	15.68	1.77
12.39	359.1486	C_20_H_22_O_6_	—	1.93	—
13.1	341.1382	C_20_H_20_O_5_	0.57	5.39	7.97
13.52	417.1542	C_22_H_24_O_8_	—	2.38	—
14.32	343.1536	C_20_H_22_O_5_	—	20.79	—
14.62	523.196	C_29_H_30_O_9_	1.35	2.79	—
15.39	507.2011	C_29_H_30_O_8_	2.36	27.65	9.85
16.54	299.2004	C_20_H_26_O_2_	—	—	1.12
17.18	319.2265	C_20_H_30_O_3_	3.81	—	1.55
17.75	301.2159	C_20_H_28_O_2_	1.6	—	0.9
18.18	315.1952	C_20_H_26_O_3_	0.47	—	0.59
18.41	289.252	C_20_H_32_O_2_	—	0.35	—
19.74	301.2159	Dehydroabietic acid	1.93	0.54	1.62
20.62	No mass signal	Overlapping with isopimaric acid	21.19	—	—
20.69	303.2314	Isopimaric acid	35.51	6.06	43.76
21.29	271.2418	C_20_H_30_	2.17	—	—
21.35	289.2522	C_20_H_32_O	5.91	—	—
21.44	273.2573	C_20_H_32_	—	—	4.42
21.77	273.2574	C_20_H_32_	—	0.49	—
22.23	287.2367	C_20_H_30_O	11.64	0.52	0.55
		**Total**	**91.3**	**89.29**	**88.3**

**Table 4:** Size exclusion chromatography of the resins, where *M*_n_ represents the number average molecular weight, *M*_w_ represents the weight average molecular weight and *Đ* represents the polydispersity index

Resin	*M*_n_ (g mol^−1^)	*M*_w_ (g mol^−1^)	*Đ*
Atlas cedar	301	533	1.77
Larch	316	526	1.66
Spruce (Graz)	318	596	1.87
Spruce (Linz)	307	582	1.89

For all the resins investigated in this study (Atlas cedar, larch, and spruce), we investigated the dielectric film surface *via* contact angle measurement, AFM, and KPFM.

**Fig. 4:**
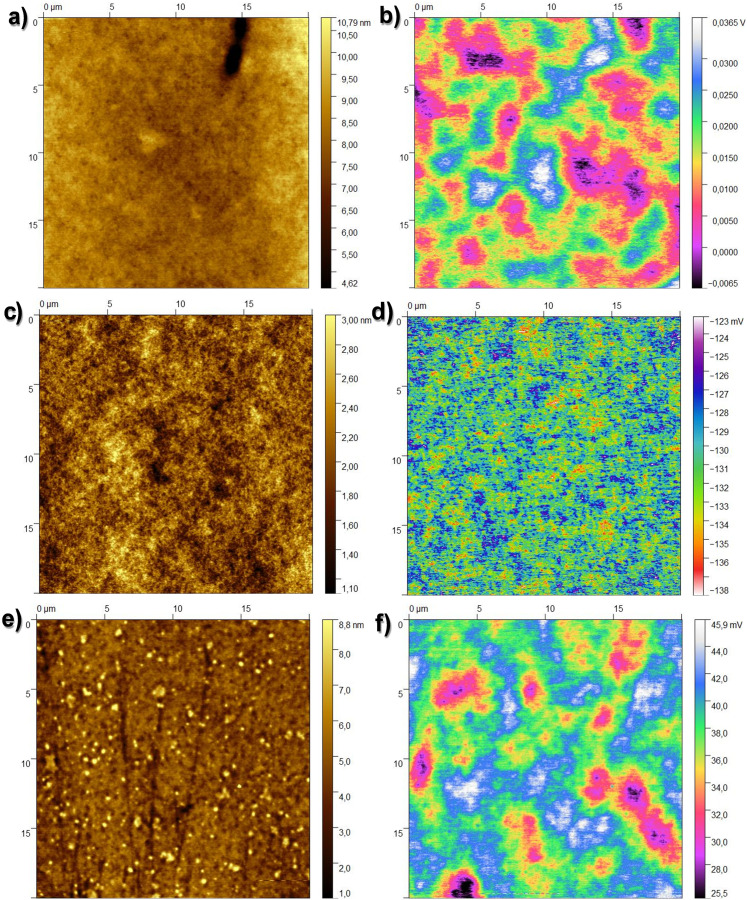
(a) 20 × 20 μm^2^ topography image of the investigated larch resin surface, height rms roughness 0.71 ± 0.30 nm; (b) corresponding surface potential map, CPD rms value 6.49 ± 1.52 mV; (c) 20 × 20 μm^2^ topography image of the investigated Atlas cedar resin surface, height rms roughness 0.3 ± 0.01 nm; (d) corresponding surface potential map, CPD rms value 2.98 ± 0.41 mV; (e) 20 × 20 μm^2^ topography image of the investigated spruce resin surface, height rms roughness 1.23 ± 0.25 nm; and (f) corresponding surface potential map, CPD rms value 4.55 ± 1.94 mV.

**Fig. 5:**
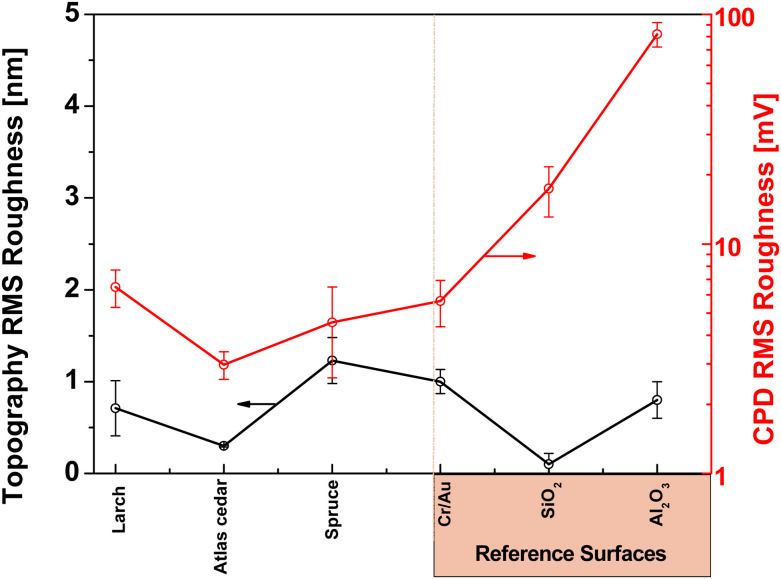
Topography RMS roughness (left axis black line) and contact potential difference RMS roughness of larch, Atlas cedar and spruce, relative to reference surfaces (gold-capped chromium, silicon dioxide and aluminum oxide).

Fig. 4a presents the typical surface morphology observed for the larch samples. The RMS roughness of the spin-coated larch surface was determined to be essentially flat, with a height roughness of 0.71 ± 0.3 nm. The corresponding surface potential map is shown in Fig. 4b. The main features observed in the KPFM image of larch resin films correlated to higher and lower regions of the resin layer, most likely originating simply from the probe being closer and further from the grounded electrode underneath the resin layer, respectively. The CPD RMS roughness was found to be 6.49 ± 1.52 mV. Considering the smaller scale images and focusing on the flat surface yields, the CPD variation was within the error bar of the system. We also performed surface investigation of Atlas cedar and spruce resins *via* contact angle measurement, AFM, and KPFM. The surface roughness of the spin-coated Atlas cedar resins was found to be comparable to that of polished Si wafers (*i.e.*, ∼0.3 nm, see Fig. 4c), whereas the surface potential of the Atlas cedar resin film was similar to the other analyzed resins, *i.e.*, 2.98 ± 0.41 mV. In fact, the spatial variations of the CPD were found to be almost negligible on the scale of 20 μm. The surfaces of the spruce resin films were atomically smooth and comparable to that of SiO_2_ formed *via* the wet thermal oxidation of Si chips, with a root mean square height of 1.23 ± 0.25 nm. The spatial variations of the CPD values of spruce films were found to be very similar to most of the other resins investigated in this study, *i.e.*, 4.55 ± 1.94 mV. A compilation of the results from the AFM and KPFM investigations for the analyzed resins is presented in Fig. 5. Also, the results are compared with two well-known inorganic dielectrics, silicon dioxide and aluminum oxide, as well as the surface of plain gold (*i.e.*, a film of Cr/Au), respectively.

**Fig. 6:**
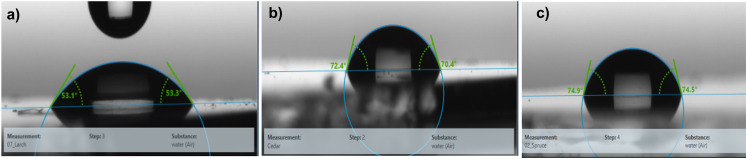
Water droplet contact angle of (a) larch resin; (b) Atlas cedar resin; and (c) spruce resin.

The water droplet contact angle measurement for larch, Atlas cedar and spruce revealed that they possessed hydrophilic surfaces (see Fig. 6). The water droplet contact angle for the larch resin, as displayed in Fig. 6a, was 53.1° ± 2.4°. The contact angle of the same resin with diiodomethane (not shown in the figure) was 43.1° ± 3°. The deviation in both quantities shows the spread of the measurement values of at least three droplets (left and right). The total surface energy, 56.5 mN m^−1^, was divided into a disperse component of 38 mN m^−1^ and a polar component of 18.5 mN m^−1^. The water droplet contact angle of the Atlas cedar resin was 70.4° ± 3.4°, as displayed in Fig. 6b. The contact angle of the same resin with diiodomethane (not shown in the figure) was 35.5° ± 2.5°. The deviation in both quantities shows the spread of the measurement values of at least three droplets (left and right). The total surface energy, 47.5 mN m^−1^, was divided into a disperse component of 41.8 mN m^−1^ and a polar component of 5.7 mN m^−1^. The water droplet contact angle of the spruce resin, as displayed in Fig. 6c, was 74.5° ± 2.1°. The contact angle of the same resin with diiodomethane (not shown in the figure) was 37.2° ± 1.2°. The deviation in both quantities shows the spread of the measurement values of at least three droplets (left and right). The total surface energy, 46.3 mN m^−1^, was divided into a disperse component of 41 mN m^−1^ and a polar component of 5.3 mN m^−1^.

**Fig. 7:**
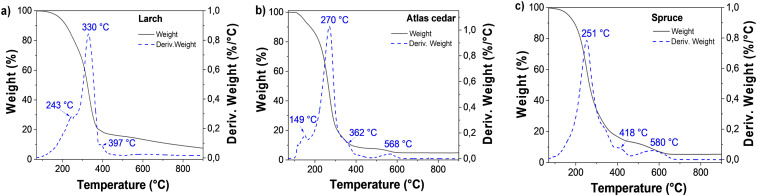
Thermogravimetric analysis of (a) larch, (b) Atlas cedar and (c) spruce.

The larch resin sample was analyzed by TGA under a nitrogen atmosphere (see Fig. 7a), and the first weight loss was detected below 120 °C (0.51%), which is attributed to possible traces of adsorbed water. The second main weight loss was centered at 330 °C; however, decomposition was also detected at the temperature of 243 °C. Here, the sample lost approximately 83% of its weight up to 450 °C. Another decomposition peak was detected at a temperature of 397 °C. From 450 °C to 650 °C, 4.32% weight loss was observed; similarly, up to 900 °C, ∼4.7% weight loss was also detected. A residual weight of 7.7% was detected, which is attributed to the incomplete decomposition of the sample.

Atlas cedar resin was analyzed by TGA (see Fig. 7b). The first transition below 120 °C showed a weight loss of 1.22%, which is attributed to the loss of water adsorbed from the environment, possibly occurring on the surface of the sample. The second step and more pronounced weight loss was centered at 270 °C (carrying approximately 91% weight loss). In this range (120–450 °C), two decomposition temperatures (at 149 °C and 362 °C) were detected. Moreover, the sample lost *ca.* 3% weight after 450 °C, with the respective peak centered at approximately 568 °C. Up to 900 °C, the very small weight loss of approximately 0.015% was detected, leaving a residual unburned weight of approximately 5%, which is attributed to the incomplete decomposition of the sample. In the case of spruce resin (see Fig. 7c), the adsorbed water was evaporated and the first weight loss was detected below 120 °C (0.94%). The second and more pronounced weight loss was centered at 251 °C, where the sample lost approximately 85% of its weight between 120 °C and 450 °C. Another decomposition peak was observed at a temperature of 418 °C, followed by a third considerable weight loss up to 650 °C of approximately 8.3%. The final step between 650 °C and 900 °C showed a minor loss of approximately 0.08%, leaving approximately 6% of residual weight.

**Fig. 8:**
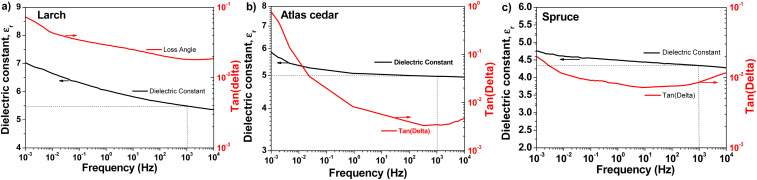
Impedance spectra of films of the investigated resins in metal–insulator–metal (MIM) structures with aluminum top and bottom electrodes. (a) Larch, (b) Atlas cedar, and (c) spruce measured in the frequency window of 10 kHz to 1 MHz. The dielectric constant for the three resins was extracted using the capacitance value measured at 1 kHz.

For the entire set of dielectric measurements (impedance spectroscopy and breakdown field), a metal–insulator–metal (MIM) configuration was used. In this investigation, each resin was spin-coated onto a metal bottom electrode, and subsequently covered with a metal top electrode. We fabricated 4 slides, consisting of 4 MIM structures on each slide for each of the resins studied herein. Subsequently, we provide the results of one example of the MIM for each resin. The resulting films were measured by profilometry in the vicinity of the MIM cross and the resulting thicknesses were 105 nm for Atlas cedar, 110 nm for larch, and 465 nm for spruce. In all three materials (see Fig. 8), we observed a very uniform capacitance across the measured frequency range, and the loss angle (tangent delta) exhibited no relaxation behavior within this range. Both observations indicate the very high purity of the dielectric film with excellent dielectric performance. This is counterintuitive, considering that the resins were simply collected from the host trees, solubilized in pure ethanol and simply filtered through a hydrophilic filter.

**Fig. 9:**
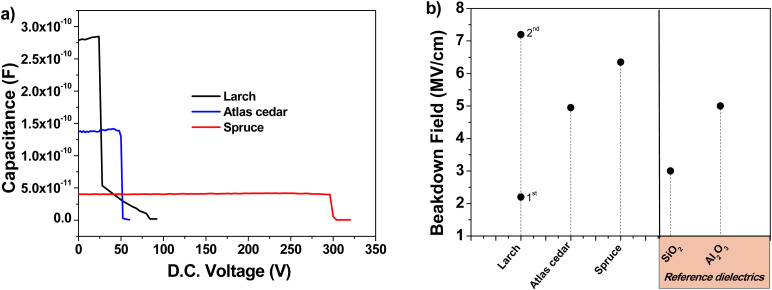
Breakdown field evaluation of the analyzed resins: (a) capacitance *vs.* applied D.C voltage showing a sharp decline in capacitance, indicating dielectric breakdown. (b) Breakdown field of the resins in comparison to the classic inorganic dielectrics SiO_2_ and Al_2_O_3_.

In the case of each material, to establish its dielectric constant, we considered its capacitance at 1 kHz. The calculated dielectric constants were 5.4 for larch, 5.0 for Atlas cedar and 4.3 for spruce. The standard deviation of the dielectric constant for the 16 MIM samples analyzed was ±0.4 for larch and Atlas cedar, and ±0.3 for spruce. In addition, the breakdown voltage of the materials was determined by coupling the impedance analyzer to a DC voltage booster with the possible voltage window of ±500 V, and the results of this study for one particular MIM structure for each resin are presented in Fig. 9. The thickness, dielectric constant, breakdown voltage, and breakdown field of the investigated materials are summarized in Table 5.

**Table 5:** Dielectric constant, breakdown voltage and breakdown field for the analyzed resins

Material	Thickness/nm	Dielectric constant	Breakdown voltage/V	Breakdown field/MV cm^−1^
Larch	110	5.4 ± 0.4	25 (1st stage)	2.3
			80 (2nd stage)	7.3
Atlas cedar	105	5.0 ± 0.4	52	4.95
Spruce	465	4.3 ± 0.3	296	6.35

Given that the 16 MIM structures under analysis were all made of resin films that were cast from the same solution and rotated at the same speed, there was very little variation in the film thickness. To generate a reliable Weibull distribution of the breakdown field values, one should follow a traditional breakdown field analysis that accounts for the fluctuation in the dielectric thickness. Nevertheless, this topic will be part of our future investigation on the breakdown field of plant resins. The spruce and Atlas cedar resins broke suddenly (the measured capacitance dropped abruptly to a negative value) when the appropriate voltage was reached (see Fig. 9a). However, the larch film broke in two stages, and thus Fig. 9b presents two separate breakdown field values. Considering that the breakdown field of anodized aluminum (*i.e.*, Al_2_O_3_) is likewise not greater than 5 MV cm^−1^, the dielectric strength values approaching or exceeding 5 MV cm^−1^ for all the examined resins are notable^81^ (see Fig. 9 and Table 5). These values are consistent with the trend observed for other investigated tree or animal resins reported previously by our group.^82–84^ In comparison, synthetic polymer resins such as benzocyclobutene (BCB) have a breakdown field or dielectric strength of no more than 4.5 MV cm^−1^, while the stated breakdown field for typical low-*k* polymer dielectrics is between 1 and 2 MV cm^−1^. Generally, it is known that the breakdown voltage is dependent on the film thickness, as shown by the equation *V* = *At*^2/3^, where *t* is the film thickness and *A* is a material constant. Consequently, the dielectric strength decreases as the dielectric thickness increases. Accordingly, as shown elsewhere,^85^ the breakdown field values reported in this work may need to be reexamined in a more systematic investigation because they represent only a specific example, and are particular for the thickness of the dielectric films used.

**Fig. 10:**
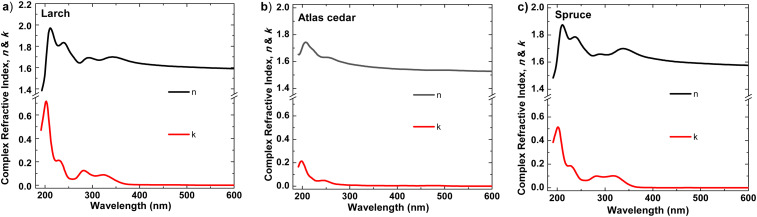
Complex refractive index of (a) larch, (b) Atlas cedar, and (c) spruce.

For the determination of the complex refractive index, *n* and *k*, were obtained by spectroscopic ellipsometry in the spectral range of 195 nm to 1685 nm. The resulting graphs can be seen in Fig. 10. All the investigated resins show multiple optical transitions in the UV range. However, they are basically transparent within the visible and near IR range. The tail absorptions extend into the visible range, giving the resins a slightly tainted appearance in thick layers. The resins are isotropic, and their real refractive indices are in the range of 1.5 to 1.6, which are similar to that of float glass.

### 3.2 Transistor measurements

**Fig. 11:**
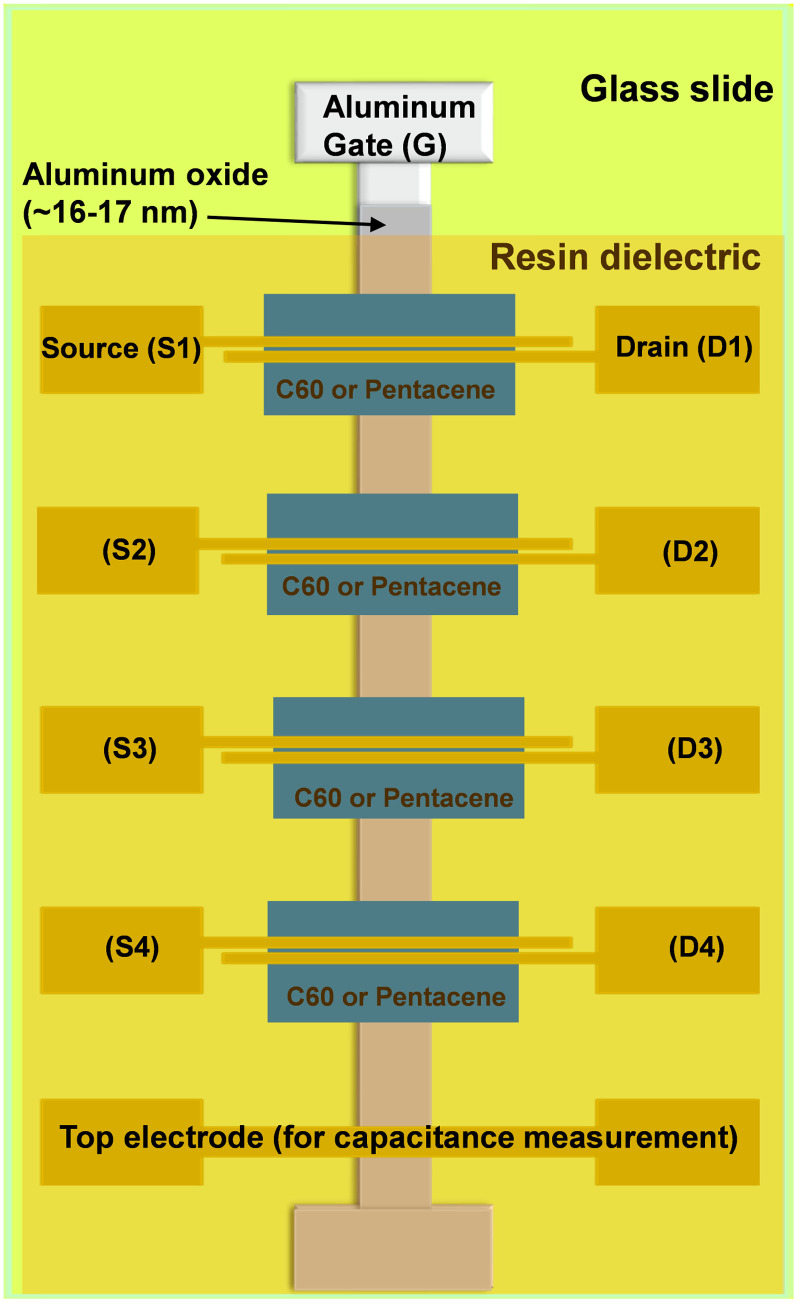
OFET design utilized in this work. We employed a series of evaporations using three distinct shadow masks: a gate mask for the development of the gate electrode; a semiconductor mask for the development of four distinct patches of the organic semiconductor (*i.e.*, either pentacene or C_60_); and a source–drain mask for the development of four pairs of top source and drain contacts and one continuous contact for the dielectric specific capacitance measurement. Four separate OFETs, each identified appropriately in the schematic, are present in the finished structure on a single glass slide. The OFET channel dimensions were length, *L* = 25 μm and width, *W* = 2 mm.

**Fig. 12:**
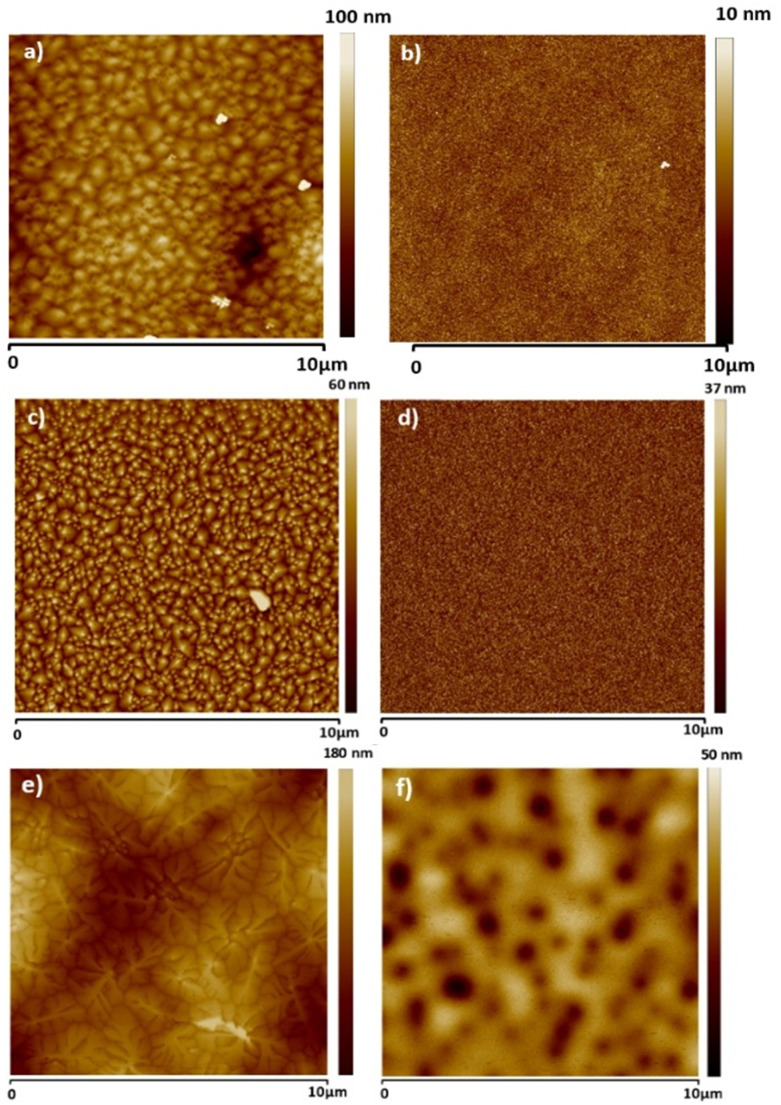
AFM scans of the semiconductors grown on the dielectric capping layer. (a) Pentacene on larch, (b) C_60_ on larch; (c) pentacene on Atlas cedar, (d) C_60_ on Atlas cedar; (e) pentacene on spruce and (f) C_60_ on spruce.

All three resins investigated in this study were tested as a capping layer of the aluminum oxide dielectric in OFETs. Two different semiconductors (pentacene and C_60_) were selected to obtain p- and n-type operating field effect transistors, respectively (see Fig. 11). For each resin used in this investigation, two batches of transistors were made, one with pentacene and the other with C_60_. Given that our fabrication mask has six glass slide positions, similar to that schematically shown in ref. 84, and that each slide contains four transistors, as shown in Fig. 11, a total of 48 OFETs was made for each resin and pentacene and likewise 48 OFETs for each resin and C_60_. In addition to the classic transfer and output characteristics recordings, bias stress measurements and stability measurements of the fabricated transistors were performed. Fig. 12 depicts the AFM images of the three resins with either pentacene or C_60_ grown on top. Fig. 12a and b present the topography of the semiconductors (pentacene and C_60_) grown on the films of larch. Both the pentacene and C_60_ grains are uniform in size; however, pentacene grew in aggregates with a typical size of 400 to 650 nm, whereas the grains of C_60_ are very small (in the range of 10 nm). In the AFM images of Atlas cedar (pentacene in Fig. 12c and C_60_ in the panel of Fig. 12d), it can be seen that the two morphologies also significantly differ. Pentacene grew in a uniform size of 300 to 500 nm, while C_60_ grew in rather small grains (10 to 30 nm). On spruce (pentacene in the panel of Fig. 12e and C_60_ in the panel of Fig. 12f), the grains of both semiconductors are uniform in size. However, pentacene grew in much larger grains than on larch and Atlas cedar (1.5 to 3 μm dendritically shaped aggregates), whereas the grains of C_60_ again have a uniform size of 10 nm.

**Fig. 13:**
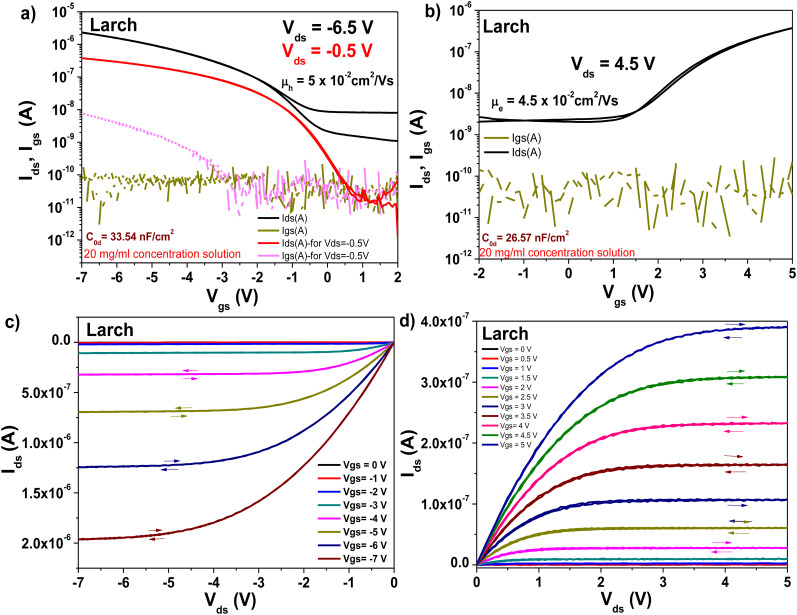
Transistor measurements of Larch resin on ∼17 nm thick Al_2_O_3_ gate with pentacene and C_60_ as organic semiconductors. (a) Transfer curve of the OFET using pentacene as the organic semiconductor. (b) Transfer curve of the OFET using C_60_ as the organic semiconductor. (c) Output curve of the OFET using pentacene as the organic semiconductor. (d) Output curve using C_60_ as the organic semiconductor.

**Fig. 14:**
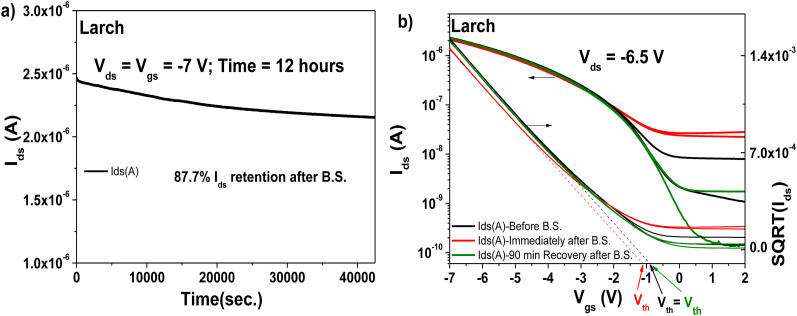
OFET with Larch resin and pentacene semiconductor evaluated for bias stress stability: (a) bias stress measurement curve after applying constant voltages to the OFET device, *V*_ds_ = *V*_gs_ = −7 V for 12 hours. (b) Recovery of the transistor characteristics after the bias stress measurements.

The transistors produced with larch resin as the capping layer on 10 V anodized Al_2_O_3_ (*i.e.*, ∼16–17 nm thick aluminum oxide) showed hysteresis-free behavior both in transfer and output characteristics (with both semiconductors, see Fig. 13, panels a–d). The dielectric behavior was characterized by low leakage in the range of 100 pA through the whole measurement range (*i.e.*, −2 to 5 V for the fullerene semiconductor and 2 to −7 V for pentacene as the semiconductor). In the case of both semiconductors, the mobility was calculated to be in the range of 0.05 cm^2^ V^−1^ s^−1^. The subthreshold swing of the two semiconductors was 0.8 V dec^−1^ for pentacene and 1.9 V dec^−1^ for the fullerene film. In the case of the normalized subthreshold swing values, we obtained 41.42 V nF cm^−2^ dec^−1^ for pentacene and 50.47 V nF cm^−2^ dec^−1^ for C_60_. In general, the normalized subthreshold swing is the more convenient method to compare the performance of organic semiconductors in OFET devices when deposited on various dielectrics or on dielectrics of different thicknesses.^11,86^ For both semiconductors, the subthreshold swing and the normalized subthreshold swing were in the same range, despite the slight difference in the thickness of the larch dielectric, which is visible in the small discrepancy of the normalized capacitances, C_0d_, listed as insets in the transfer characteristic graphs. A characteristic of the larch resin was that it induced a relatively high OFF level in the organic transistor characteristics, possibly because of the tendency of the dielectric material to charge the semiconductor in the OFF state. As shown in Fig. 13a and b, the OFF level of both p- and n-type devices was greater than 1 nA. Nevertheless, despite this high OFF level, the transistors showed virtually hysteresis-free characteristics. In addition to the classic transistor characterization measurements, bias stress investigations of the fabricated OFET with pentacene as semiconductor were performed, as presented in Fig. 14. We stressed the device at the maximum voltage (−7 V), while the voltage for both source and drain were kept constant for 12 h stress time (Fig. 14a). The transfer characteristics were measured at the beginning of the experiment as well as immediately after releasing the electrical stress. The larch-based OFET device retained ∼87.7% of its initial *I*_ds_ after the stress time of 12 h, which is a remarkable value that ranks high among the best reported combinations of organic dielectrics and semiconductors.^2,10,87,88^ We continued to measure the recovery curve of the devices with 15 min increment between measurements (Fig. 14b), but for simplicity and to avoid cluttering the graph, we only show the transfer curve, where full or nearly full recovery was measured. In the case of larch, the ON level of the drain current, as well as the threshold voltage recovered after 90 min relaxation after the bias stress time of 12 h.

**Fig. 15:**
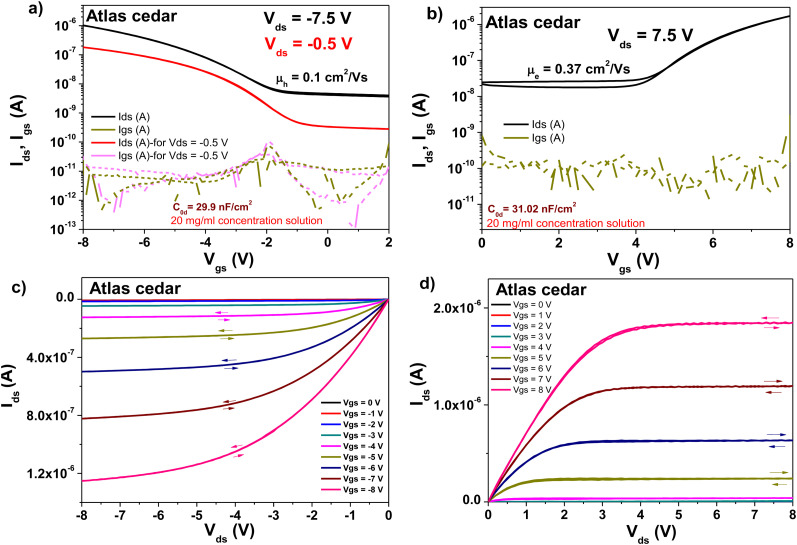
Transistor measurements of Atlas cedar resin on 10 V anodized aluminum gate electrode (*i.e.*, ∼16–17 nm thick aluminum oxide, Al_2_O_3_) with pentacene and C_60_ as the organic semiconductor: (a) transfer curve of the OFET using pentacene as organic semiconductor. (b) Transfer curve of the OFET using C_60_ as the organic semiconductor. (c) Output curve of the OFET using pentacene as the organic semiconductor. (d) Output curve using C_60_ as the organic semiconductor.

The transistor, which was fabricated with a capping layer of Atlas cedar resin on aluminum oxide dielectric, also exhibited hysteresis-free behavior in both its transfer and output characteristics (Fig. 15, panels a–d). Similar to the case to larch resin, the Atlas cedar resin-based OFETs displayed a high OFF level for both the p- and n-type devices, this time in the range of 10^−8^ A or higher. In the case of the field effect mobilities, values of 0.1 cm^2^ V^−1^ s^−1^ for pentacene and 0.37 cm^2^ V^−1^ s^−1^ for C_60_ were calculated, with both OFET devices working at an applied gate voltage of ±8 V. For the subthreshold swing, we obtained a value of 2.4 V dec^−1^ for pentacene and 2.2 V dec^−1^ for fullerene, which are basically in the same range, a fact not surprising given the similarity of the two transfer curves. The normalized subthreshold swing was around the same value too, *i.e.*, 23.5 V nF cm^−2^ dec^−1^ for the pentacene-based device and 21.8 V nF cm^−2^ dec^−1^ for the device with fullerene, given that the two devices also have a similar specific capacitance (displayed as the inset of the two transfer characteristics curves). The higher mobility values of the Atlas cedar resin-based OFETs compared to their larch resin-based counterparts is a direct consequence of the inherently high OFF level for the Atlas cedar-based transistors, which generates a high value for the slope (*Δ*) in the mobility formula. If hypothetically the OFF level current would have been in the range of microamps, the recorded field effect mobilities would have surely surpassed the value of 1 cm^2^ V^−1^ s^−1^, as shown before.^89^

**Fig. 16:**
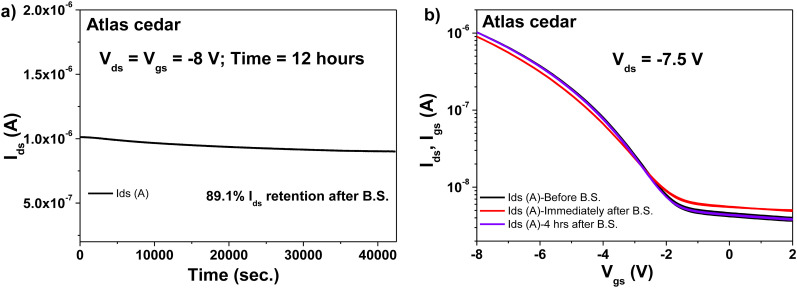
OFET with Atlas cedar resin and pentacene semiconductor evaluated for bias stress stability: (a) bias stress measurement curve after applying constant voltages to the OFET device, *V*_ds_ = *V*_gs_ = −8 V for 12 h. (b) Recovery of the transistor characteristics after the bias stress measurements.

We performed the bias stress investigations with the transistor consisting of an Al_2_O_3_ dielectric, an Atlas cedar resin capping layer, and pentacene as the semiconductor. The transistor device was stressed for 12 h at the maximum allowed voltage before breaking would occur (−8 V). The device showed very good bias stress resistance, with ∼89% of the drain current retained after 12 h of stress (Fig. 16a), which similar to the larch-based OFET presented above, represents a remarkable result. The transfer characteristic was fully recovered after 4 h relaxation after the stress (see Fig. 16b).

**Fig. 17:**
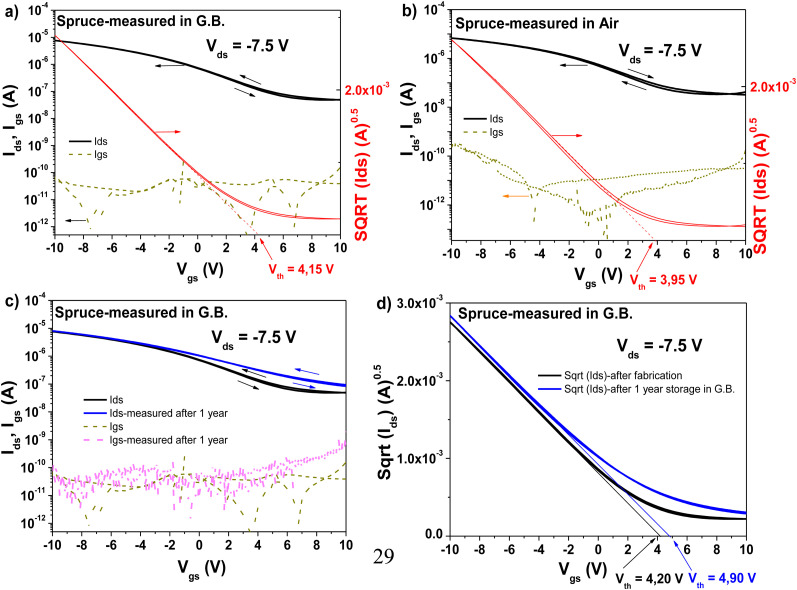
Spruce resin based OFET characteristics with pentacene semiconductor measured (a) in a glove box and (b) in ambient air. (c) Comparison of the OFET characteristics measured immediately after fabrication and 12 months of storage in a glove box under nitrogen. The OFET device was never remeasured during the 12-month span. (d) Device shown in panel (c), with the vertical axis expressed as square root of (*I*_ds_), showing the drift of the threshold voltage, *V*_th_, during the storage time.

Similar to the other two resins investigated in this study, spruce resin was also implemented as a capping layer interface to aluminum oxide, with pentacene and C_60_ semiconductors. When interfaced to pentacene semiconductor, the OFET displayed a very high OFF level in the transfer characteristics, in the range of 10^−7^ A (see Fig. 17). In contrast, when interfaced with C_60_, the OFF level was one order of magnitude lower, *i.e.*, 2 × 10^−8^ A, resulting in a low ON–OFF ratio OFET (data not shown). Because of the very high OFF level of both the n-type and p-type devices, we were not able to record the bias stress for spruce. In the case of bias stress for the aluminum oxide-spruce-pentacene OFET, the transfer characteristic turned into a perfect horizontal line at the end of the 12 h bias stress, and this horizontal *I*_ds_*vs. V*_gs_ line never recovered during the next four days when the system was allowed to relax. In this respect, thus far, spruce remained the only resin in the entire set of Pinaceae resins for which this type of test could not be completed.^83,84^ To account for the stability of the spruce-based OFET device, we instead measured the device with the pentacene semiconductor in a glove box and compared it with the measurement obtained by employing a probe station in ambient air. The cables used in the experiment were identical to remove any possible discrepancy in the obtained results due to the difference in impedance of the measurement cables. The respective comparison is presented in Fig. 17, proving the relatively good stability of the interface between the spruce resin and the pentacene semiconductor, with the threshold field values differing by only 0.1 V. In addition, we remeasured the transfer curve presented in Fig. 17a after one year storage in a glove box, and the results are displayed in Fig. 17c and d, proving the good stability of the spruce resin interface with pentacene. In this respect, a drift in the threshold voltage of ∼0.7 V was recorded.

**Fig. 18:**
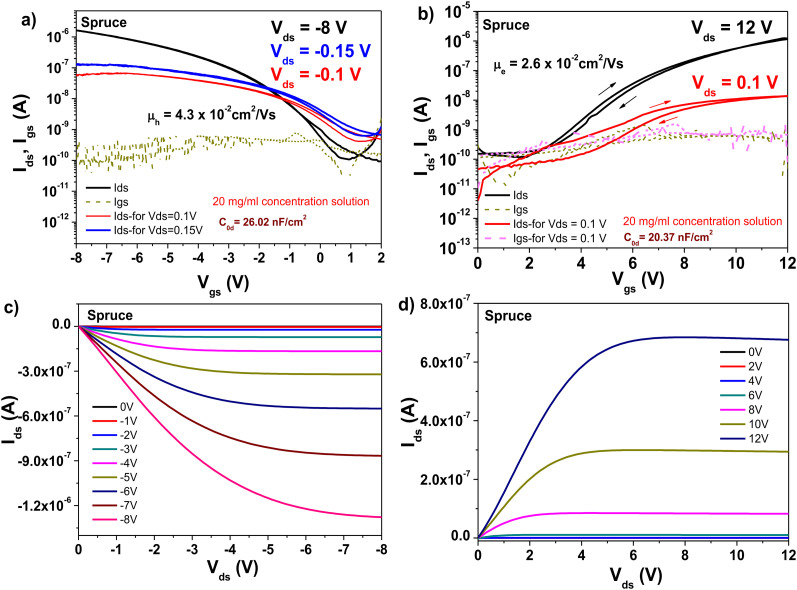
Transistor measurements of spruce resin on 10 V anodized aluminum gate electrode (*i.e.*, ∼16–17 nm-thick aluminum oxide, Al_2_O_3_) with pentacene and C_60_ as the organic semiconductor: (a) transfer curve of the OFET using pentacene as the organic semiconductor displaying the minimum drain–source voltage able to increase the characteristics, *i.e.* 150 mV. (b) Transfer curve of the OFET using C_60_ as the organic semiconductor. (c) Output curve of the OFET using pentacene as the organic semiconductor. (d) Output curve using C_60_ as the organic semiconductor.

The bias stress event of spruce prompted us to investigate one more spruce resin for the fabrication of OFETs. The new resin was collected from a tree in the vicinity of the city of Linz, Austria, which was only characterized *via* a size exclusion chromatography experiment, as presented in Table 4. Although the number average molecular weight (*M*_n_) and weight average (*M*_w_) values differ slightly, as presented in Table 4, the new resin also displayed hysteresis-free behavior in both the transfer and output characteristics of OFETs when interfaced with pentacene, and small hysteresis in the respective characteristics when interfaced with C_60_ (see Fig. 18). Basically, these results are identical with that of the original spruce resin sample collected from a tree growing in the vicinity of the city of Graz, Austria. Nevertheless, the OFF level of the new resin had lower values by one to two orders of magnitude, as clearly shown in Fig. 18a and b. Interestingly, in the case of the field effect mobilities, we obtained relatively similar values for both semiconductors following the identical trend as the respective values obtained for the original resin, *i.e.*, in the range of 4.3 × 10^−2^ cm^2^ V^−1^ s^−1^ for the pentacene- and 2.6 × 10^−2^ cm^2^ V^−1^ s^−1^ for the C_60_-based devices. In the case of the original resin (its transfer curves with pentacene are shown in Fig. 17), these values were 3.9 × 10^−2^ cm^2^ V^−1^ s^−1^ for the pentacene- and 4.6 × 10^−2^ cm^2^ V^−1^ s^−1^ for the C_60_-based devices, respectively. A subthreshold swing of 0.85 V dec^−1^ for pentacene and 1.7 V dec^−1^ for C_60_ were recorded for the fabricated OFETs with the new resin. The calculated values of the normalized subthreshold swing were 22.1 V nF cm^−2^ dec^−1^ for the pentacene-based device and 44.1 V nF cm^−2^ dec^−1^ for fullerene, respectively. The minimum drain–source (*V*_ds_) voltage that could run the spruce resin-based OFET was 150 mV, as shown in Fig. 18a, given that at an applied drain–source voltage of 100 mV, the curve still pitched down in full saturation.

The entire set of OFET parameters for the three analyzed resins in this study is summarized in Table 6. Inspecting Table 6, one can observe that the three resins investigated showed a relatively uniform performance in OFETs, with the values of the critical parameters that characterize the respective devices being in the same range. Thus, it is very difficult to clearly state which one is more or less efficient than other when employed as the capping layer for the aluminum oxide dielectric. Even when collected from trees that are situated several hundred kilometers away, the resins showed similar trends and performances, as demonstrated by the spruce resin collected from two distant trees, which had almost identical polydispersity index values, *Đ* (*i.e.*, 1.89 *vs.* 1.87). Obviously, the number average molecular weight (*M*_n_) and weight average (*M*_w_) values may differ even when comparing resin samples collected from the same tree, given the unknown age of the respective material or the position on the tree stem (*i.e.*, in direct sun exposure or on the north side, where the UV-exposure is negligible). In this case, we performed size exclusion chromatography measurements on a third spruce sample, which was collected from a different deposit situated on the same tree in Linz for which the resin was discussed above as part of the OFET devices in Fig. 18. The obtained values for the *M*_n_, *M*_w_, and *Đ* parameters were 311, 565, and 1.81 respectively, for the third resin, which are not significantly different than the values of the two other spruce resins investigated herein. Interestingly, also, all the resins examined in this study generated high OFF level values in the transfer characteristics of OFETs, and hysteresis-free behavior especially when interfaced with pentacene. Thus, these resins seem to be an excellent capping layer for inorganic dielectrics such as aluminum oxide, which show great reactivity especially towards electron transport.^90,91^

**Table 6:** OFET parameters for the fabricated devices: threshold voltage (*V*_th_), specific capacitance (*C*_0d_), ON–OFF ratio (*I*_ON_/*I*_OFF_), field effect mobility (*μ*), and subthreshold swing (*S*_SW_)

Resin	OFET parameters									
	With pentacene					With C_60_				
	*V*_th_ (V)	*C*_0d_ (nF cm^−2^)	*I*_ON_/*I*_OFF_	*μ*_h_ (cm^2^ V^−1^ s^−1^)	*S*_SW_ (V dec^−1^)	*V*_th_ (V)	*C*_0d_ (nF cm^−2^)	*I*_ON_/*I*_OFF_	*μ*_e_ (cm^2^ V^−1^ s^−1^)	*S*_SW_ (V dec^−1^)
Larch	−1.4	33.54	1966	5 × 10^−2^	0.8	2	26.57	176	4.5 × 10^−2^	1.9
Atlas cedar	−1.8	29.9	250	0.1	2.4	4.5	31.02	97	0.37	2.2
Spruce	−0.4	26.02	1.5 × 10^4^	4.3 × 10^−2^	0.85	5	20.37	1 × 10^4^	2.6 × 10^−2^	1.7

Also, the nearly hysteresis-free electric performance of all the materials under investigation in this study is noteworthy and impressive, particularly when they were interfaced with the p-type semiconductor pentacene. This was also noted for fir and pine Pinaceae resins, as recently published by us.^83,84^ Compared to other traditional dielectrics used as a capping layer for aluminum oxide, such as parylene-C, divinyltetramethyldisiloxane-bis(benzocyclobutene) (BCB), low density polyethylene, adenine, shellac, lignin and cellulose in combination with aluminum oxide,^54,82,92–97^ the examined Pinaceae resins reported herein perform exceptionally well.

## 4. Conclusion

In this study, we investigated three resins originating from widespread Pinaceae trees and applied them as dielectrics in organic field effect transistors. If a single advantage of these resins has to be chosen, it is their abundant availability at virtually no cost. Spruce is the most widespread tree in the Eurasian forests, and has extensive industrial applications in the production of paper and nanocellulose fibers. Alternatively, larch is not as abundant; nevertheless, it is the material of choice for the production of floor planks, especially in Europe. Atlas cedar is a majestic tree of optically pleasant crown appearance, which is used extensively as an ornamental tree in many parks and residential areas in nearly all cities in Europe. In this work, we analyzed the resins collected from the above-mentioned living trees *via* a large set of experimental techniques to understand their composition, processing and film-forming characteristics, film surface performance in terms of rms roughness and contact potential difference fluctuations, optical behavior, and finally dielectric and electronic properties when employed as a thin capping layer for aluminum oxide in organic field effect transistors. We demonstrated that their performance is arguably similar, and the obtained results are not very different. Considering that (i) these materials are free of charge, representing non-commercialized residues of the industrial trees processed for a plethora of applications, (ii) they can be easily processed using non-toxic solvents such as ethanol and potentially many other alcohols and do not require any purification step except filtration, and (iii) they are completely non-toxic and even have medicinal properties, these resins may represent materials of choice for the future development of “green” and sustainable electronic applications. Although intense efforts have been devoted to the synthetic field of research to provide materials that are stable during operation, future consumable electronics may definitely require abundant and sustainable biomaterials with minimal cost, such as those exemplified in this study, that do not pose a threat to the environment.

## Conflicts of interest

The authors do not declare any conflict of interest.

## Data Availability

Data are available from the authors upon request.
